# Mindfulness-Based Intervention in Schools: Addressing Social Media Burnout and Enhancing Well-Being in Adolescents

**DOI:** 10.3390/children12070826

**Published:** 2025-06-23

**Authors:** Süleyman Ünlü, Kıvanç Uzun, Gökmen Arslan

**Affiliations:** 1 Department of Turkish Education, Faculty of Education, Uşak University, Uşak 64200, Türkiye; 2 Department of Psychological Counselling and Guidance, Faculty of Education, Uşak University, Uşak 64200, Türkiye; 3 Department of Psychological Counselling and Guidance, Faculty of Education, Burdur Mehmet Akif Ersoy University, Burdur 15030, Türkiye

**Keywords:** social media burnout, subjective well-being, mindfulness-based intervention, adolescents

## Abstract

**Background:** This study examined the effectiveness of a mindfulness-based psychoeducational intervention in addressing social media burnout and enhancing subjective well-being among adolescents. **Methods:** A total of 138 high school students in Türkiye were screened, and 32 were randomly assigned to either an experimental group (*n* = 16) receiving a six-week intervention or a control group (*n* = 16) receiving no treatment. The intervention included weekly sessions focused on present-moment awareness, digital detox strategies, emotional regulation, and self-compassion. Data were collected using self-report scales measuring social media burnout and subjective well-being, and were analyzed using non-parametric statistical tests, including the Mann–Whitney U test and the Wilcoxon Signed-Rank test. **Results:** The results demonstrated significant improvements in the experimental group, including reduced burnout and enhanced well-being, with the effects sustained at a 30-day follow-up. **Conclusions:** These findings suggest that school-based mindfulness programs can serve as effective and scalable tools to foster psychological resilience and digital well-being in adolescents.

## 1. Introduction

The rapid penetration of digital technologies into all areas of life has led to increased exposure to social media platforms, particularly during developmentally sensitive periods [1]. Adolescence is a critical developmental stage characterized by identity formation, the establishment of social bonds, and the development of emotional regulation skills [2,3,4]. During this period, individuals become more cognitively and emotionally receptive to environmental influences. In this context, social media serves a dual role for adolescents: while it provides a network of social interaction that can foster a sense of belonging [5], it also operates as a domain where self-perception is increasingly shaped through social comparison processes [6]. On the positive side, adolescents frequently use social media to maintain peer relationships, explore their identities, and gain access to social support—especially when such support is not available in offline contexts [7,8]. These functions may enhance feelings of connection, self-expression, and emotional closeness. However, these same platforms are saturated with mechanisms that quantify popularity and approval—such as likes, comments, and follower counts—which often promote external validation-seeking, upward social comparison, and fear of missing out (FOMO) [9,10]. As a result, even while fostering connection, social media can simultaneously erode adolescents’ self-worth and heighten emotional reactivity, especially in developmentally sensitive phases. However, external validation mechanisms embedded within social media platforms—such as likes, comments, and follower counts—may contribute to instability in adolescents’ self-worth [1,11]. This, in turn, can heighten psychological vulnerability, leading to erosion of self-efficacy and an increased risk of diminished subjective well-being [12,13]. These effects are particularly concerning in adolescence, when the development of a stable sense of self and psychological flexibility becomes critical [14,15].

Within this framework, social media burnout emerges as one of the key psychological constructs with profound implications for adolescents in the digital age. This construct comprises three core dimensions: emotional exhaustion (a sense of depletion caused by overwhelming social demands), ambivalence (conflicted feelings toward social media use, such as simultaneous attraction and aversion), and depersonalization (a psychological detachment from social interactions occurring online), as identified by Han [16]. Social media burnout is characterized by these interrelated symptoms, which result from prolonged exposure to social media interactions, and is considered a distinct psychological challenge of modern times [16]. Among adolescents in particular, a mismatch between expectations related to social media use and actual experiences often fosters feelings of frustration, inadequacy, and worthlessness [11,17]. Over time, this dissonance may erode self-efficacy, lead to chronic digital fatigue, impair cognitive focus, and increase tendencies toward social withdrawal—ultimately contributing to a significant decline in subjective well-being [9,18,19]. In particular, adolescents who experience emotional exhaustion and detachment as part of social media burnout may begin to avoid both online and offline social interactions as a form of self-protection [20]. This avoidance behavior, often driven by feelings of inadequacy or fear of judgment, has been empirically linked to increased social isolation and reduced peer engagement [21,22]. Recent empirical studies continue to affirm the association between excessive social media exposure and burnout symptoms among adolescents [23,24], yet evidence-based school interventions remain scarce. These findings underscore the need for theoretically informed frameworks that promote emotional resilience, cognitive reframing, and value-driven behavior in adolescents—a focus central to both Acceptance and Commitment Therapy (ACT) and positive psychology [14,25,26].

In parallel, the concept of subjective well-being plays a critical role in understanding adolescents’ psychological responses to social media use at a conceptual level [27]. Subjective well-being is commonly defined as a multidimensional construct comprising three interrelated components: life satisfaction (a cognitive evaluation of one’s overall life), the frequent experience of positive emotions, and the infrequent experience of negative emotions [28]. These dimensions together reflect how individuals perceive and emotionally respond to their lives. Recent studies have shown that increases in time spent on social media and the intensification of social comparisons in online environments are associated with notable declines in subjective well-being among adolescents [29,30,31]. This decline not only adversely affects internal psychological processes [32], but also creates a multi-layered vulnerability across various life domains by contributing to familial tensions, weakened social connectedness, and reduced academic performance [33,34,35].

In collectivist cultural contexts such as Türkiye—where adolescents’ identity development is strongly shaped by family obligations, interdependent self-construals, and societal expectations—the negative consequences of social media burnout may extend beyond individual distress, significantly impacting family dynamics and academic motivation [36,37]. In such societies, adolescents may feel greater emotional burden when their digital struggles interfere with their performance in family-valued domains, such as school achievement or social harmony [38]. By contrast, in more individualistic cultures, the psychological impact of social media burnout may manifest more internally (e.g., as self-esteem or identity issues), with less perceived repercussion in family-related domains [39]. Given the strong association between academic success, familial approval, and social status in such contexts [40], declines in exam performance tend to heighten parental expectations and adolescents’ perceived pressure [41], further intensifying their emotional burden and compromising subjective well-being [42]. Despite the growing prevalence of digital distress in adolescence, culturally sensitive interventions that address mechanisms like validation-seeking and emotional reactivity remain underdeveloped in the mental health literature targeting collectivist societies.

This multi-layered psychosocial vulnerability underscores a critical gap in existing research: the lack of integrative, developmentally appropriate, and school-based interventions designed to mitigate the psychological costs of digital engagement among youth. In this context, the mindfulness approach—defined as the intentional and nonjudgmental awareness of the present moment—has emerged as a promising intervention framework [43,44,45]. Mindfulness theory posits that awareness-oriented practices can interrupt maladaptive automatic thought patterns and foster emotional balance—capacities essential for resisting the pull of social comparison and external validation [15,46]. A growing body of literature provides compelling evidence that mindfulness-based interventions are effective in reducing stress levels [47], improving emotional regulation skills [48], and fostering self-compassion and empathy among adolescents [49]. As such, mindfulness not only alleviates acute stress responses, but also cultivates a core set of psychological skills that support long-term subjective well-being and psychological resilience [50,51].

When examined specifically within the context of social media burnout, mindfulness practices appear to facilitate adolescents’ recognition of their automatic behavioral patterns related to social media use, enabling them to transform these patterns through conscious and intentional responses [52]. Mindfulness skills help adolescents to become less emotionally affected by social comparison processes prevalent in online environments, disrupt the burnout cycle driven by external validation seeking, and promote more autonomous and healthy regulation of social media habits [53]. This process not only fosters increased awareness regarding digital media use, but also strengthens adolescents’ self-regulatory capacities and contributes to the restructuring of psychological resilience resources [54,55,56]. These mechanisms directly align with the positive psychology perspective, which views well-being as a skillset that can be cultivated through intentional practices and strengths-based interventions [25,57]. In this regard, mindfulness offers promising potential to help adolescents develop a more resilient internal foundation against the psychosocial risks of the digital world and to support their subjective well-being in a sustainable, long-term manner.

While mindfulness-based approaches provide a valuable foundation for reducing social media burnout and enhancing subjective well-being among adolescents [51,52], the theoretical framework of the present study extends beyond mindfulness alone. It also incorporates other prominent paradigms of contemporary psychology. In particular, positive psychology and the ACT serve as two integrative pillars underpinning the intervention model. Positive psychology aims to systematically enhance psychological well-being by focusing on individuals’ strengths, positive emotions, and the pursuit of meaning in life [57]. In the context of this intervention, elements from positive psychology were operationalized through session activities aimed at enhancing gratitude, identifying personal strengths, and fostering future-oriented hope. Within this framework, fostering the experience of positive emotions, orienting individuals toward valued life goals, and cultivating a sense of self grounded in personal strengths are considered core mechanisms that contribute to subjective well-being [25,26,58].

In parallel, the ACT framework encourages individuals to lead meaningful lives guided by personal values, not by avoiding or suppressing distressing thoughts and emotions, but by accepting them as part of the human experience [14]. The intervention incorporated ACT principles particularly through practices designed to increase psychological flexibility, such as present-moment awareness exercises, acceptance-based reframing of negative thoughts, and value clarification tasks integrated into weekly reflections. For individuals—particularly adolescents—who often struggle with negative social comparisons, fear of rejection, and feelings of inadequacy triggered by social media environments, the psychological flexibility skills promoted by ACT hold critical significance [59,60]. Accordingly, enabling adolescents to more flexibly cope with social media-induced negative thoughts and emotions, to cultivate a value-driven life orientation, and to reconstruct a sense of meaning in their lives are central aims of the present intervention program. This multidimensional intervention model therefore responds directly to the call for integrative frameworks that target the emotional, cognitive, and behavioral dimensions of digital distress among adolescents.

Grounded in this comprehensive theoretical framework, the present study aims to fill a specific research gap by experimentally testing a structured mindfulness-based program targeting social media burnout in adolescence. First and foremost, it is among the few experimental investigations to systematically evaluate the effectiveness of a mindfulness-based intervention aimed at preventing social media burnout [52,61,62]. In doing so, the study sheds light on critical dimensions such as sustainability and long-term effectiveness by tracking the impact of the intervention over time. Moreover, the development and implementation of this intervention within a collectivist cultural context (Türkiye) addresses a notable gap in the literature by exploring how social media burnout interacts with cultural dynamics. Examining the interplay between social media use and broader systems of individual and societal values aligns with contemporary approaches emphasizing the necessity of culturally sensitive psychological interventions [63,64]. Additionally, the study aims to systematically teach a set of integrated skills—including digital detox strategies, present-moment awareness practices, and the cultivation of self-compassion—within a structured and holistic intervention program. In this respect, the current study contributes both empirically and conceptually to the development of preventive school-based models aimed at enhancing adolescent well-being in the digital age.

### The Present Study

In light of this background, the primary aim of the current study was to examine the effectiveness of a mindfulness-based psychoeducational intervention in reducing social media burnout and enhancing subjective well-being among adolescents. The intervention program was designed to increase adolescents’ cognitive and emotional awareness regarding their social media use, while reducing tendencies toward external validation seeking and social comparison. Core components of the program include present-moment awareness, thought–emotion recognition, digital detox strategies, and the development of self-compassion skills. A quasi-experimental design with experimental and control groups was employed, and the effects of the intervention were evaluated using pre-test, post-test, and follow-up assessments through statistical analyses. Accordingly, the following hypotheses were tested:

**H1.**  *There will be a significant difference between the pre-test and post-test social media burnout scores of the experimental group, with post-test scores being significantly lower.*

**H2.**  *There will be a significant difference between the pre-test and post-test subjective well-being scores of the experimental group, with post-test scores being significantly higher.*

**H3.**  *The post-test social media burnout scores of the experimental group will be significantly lower than those of the control group.*

**H4.**  *The post-test subjective well-being scores of the experimental group will be significantly higher than those of the control group.*

**H5.**  *The positive effects observed in the experimental group will be sustained during the follow-up period after the completion of the intervention.*

## 2. Methods

### 2.1. Research Design

This study employed an experimental research design to examine the effects of a mindfulness-based psychoeducational intervention program on social media burnout and subjective well-being among adolescents. Experimental designs aim to test causal relationships by allowing researchers to systematically manipulate an independent variable—in this case, the mindfulness-based intervention—and observe its effects on one or more dependent variables (social media burnout and subjective well-being). Such designs typically involve assignment to experimental and control groups through matching or randomization procedures, and seek to control for the influence of extraneous variables [65]. In this study, a quasi-experimental approach with matched-pairs randomization was selected due to the natural school setting, where full randomization was not feasible. This design strikes a balance between internal validity and ecological applicability in real-world educational environments.

In the present study, pairs of participants with similar baseline levels of social media burnout and subjective well-being were formed and randomly assigned to either the experimental or control group using a lottery method. A pre-test, post-test, and follow-up measurement design was implemented to compare outcomes between the two groups. The experimental group participated in a six-session intervention program delivered once per week, while the control group did not receive any intervention and only completed the measurement instruments. Throughout the research process, intervention fidelity was maintained by employing a structured session plan and assigning a single trained facilitator for all sessions. This minimized variability in delivery and ensured standardization across participants.

### 2.2. Participants

This study was conducted with adolescent students enrolled in a public high school affiliated with the Turkish Ministry of National Education, located in an urban area in western Türkiye. The participant selection process was carefully designed to ensure the validity and reliability of the experimental design. Initial meetings were held with the school administration and the on-site psychological counselor to thoroughly communicate the study’s purpose, content, and implementation procedures. Following the receipt of official approvals, a total of 473 students attending the school were informed about the study, and voluntary participation requests were collected through an online application form.

In total, 138 students expressed interest in participating voluntarily. The Social Media Burnout Scale and the Adolescent Subjective Well-Being Scale were administered by the researcher to collect pre-test data. The inclusion criteria were as follows: (1) being a currently enrolled 9th–11th grade student, (2) having parental consent and personal assent, (3) completing all the required pre-test measures, and (4) being available to attend all six intervention sessions. The exclusion criteria included the following: (1) having a diagnosed psychological or neurological disorder requiring clinical treatment, and (2) current participation in another psychological intervention program. To ensure group equivalence at baseline, matched pairs were created based on three key criteria: (1) similar pre-test scores on the Social Media Burnout Scale and the Adolescent Subjective Well-Being Scale, (2) gender (male or female), and (3) age (within a one-year range). Each pair consisted of two students who closely resembled one another across all three criteria. Within each pair, students were randomly assigned to either the experimental or the control group using a lottery method. This procedure ensured that both groups had comparable baseline levels of social media burnout and subjective well-being.

The experimental group consisted of 9 female and 7 male students; the control group had the same gender distribution. The mean age of participants in the experimental group was 15.81 years (*SD* = 0.98), while the control group’s mean age was 15.82 years (*SD* = 0.66). Prior to the intervention, a Mann–Whitney U test was conducted to examine whether there were any significant differences between the two groups in terms of their pre-test scores. The results indicated no statistically significant differences between the groups in terms of social media burnout (*p* = 0.83) or subjective well-being (*p* = 0.94), confirming baseline equivalence prior to the intervention.

Although no a priori power analysis was conducted due to the practical constraints of school-based implementation, a post hoc power analysis was performed using G*Power version 3.1 [66] to assess the adequacy of the final sample size. Assuming a large effect size (Cohen’s *d* = 0.80), α = 0.05, and two-tailed testing, the achieved statistical power for the sample (*n* = 32; 16 per group) was calculated to be 0.59. While this value falls below the conventional threshold of 0.80, it reflects realistic limitations of field-based research in school contexts. To address this limitation, all hypothesis tests were supplemented with effect size calculations to ensure robust interpretation of the findings.

To ensure ethical compliance, approval was obtained from the Ethics Committee of the university with which the researchers are affiliated. Informed consent forms were collected from all participants and their legal guardians prior to participation. All participants and their parents were clearly informed—both verbally and in writing—about the aim, scope, and procedures of the study, including their rights as participants. Participation was entirely voluntary, and it was emphasized that students could withdraw from the study at any point without any justification or negative consequence. The collected data were anonymized, securely stored, and used solely for research purposes in accordance with data protection regulations. Ethical approval for the study was granted by the Research and Publication Ethics Committee of Social and Human Sciences at Uşak University (Protocol No: 2025.125; Approval Date: 13 March 2025), and the study was conducted in full compliance with the Declaration of Helsinki.

### 2.3. Measures

*Demographic Information Form*. A structured demographic information form was developed by the researcher to collect data regarding the personal characteristics of the participating adolescents. The form included items pertaining to participants’ gender and age. Additional demographic variables such as socioeconomic status, parental education level, or digital access were not included in the present study due to two key reasons: (1) the primary focus of the study was on the psychological outcomes of a structured intervention, rather than population-level demographic analysis; and (2) the implementation took place within a school-based setting where the standardization and brevity of data collection instruments were prioritized to minimize disruption to the academic schedule. Therefore, only age and gender—two variables directly relevant for the matched-pairs assignment process—were considered in participant profiling.

*Social Media Burnout Scale (SMBS).* The Social Media Burnout Scale (SMBS) was originally developed by Han [18] to assess individuals’ levels of social media burnout, a psychological condition resulting from prolonged engagement with social media platforms. The scale was adapted to Turkish culture by Gündoğan [67] and validated with both high school and university students. The SMBS is a self-report instrument consisting of 11 items across three subdimensions: ambivalence (4 items), emotional exhaustion (3 items), and depersonalization (4 items). In addition to subscale scores, a total social media burnout score can also be computed. Items are rated on a 4-point Likert scale ranging from (1) Almost Never to (4) Always. There are no reverse-coded items. Total scores range from 11 to 44, with higher scores indicating greater levels of social media burnout. The internal consistency of the scale is satisfactory, with a Cronbach’s alpha coefficient of 0.80 [67]. In the present study, the internal consistency of the SMBS was found to be 0.87, indicating high reliability. Example items from the scale include the following: Item 7: “Using social media causes me a great deal of stress”.; Item 11: “If I could, I would avoid social media altogether”.

*Adolescent Subjective Well-Being Scale (ASWBS).* The Adolescent Subjective Well-Being Scale (ASWBS) was developed by Eryılmaz [68] to assess the subjective well-being levels of adolescents. The ASWBS is a 15-item self-report instrument using a 4-point Likert scale ranging from (1) Strongly Disagree to (4) Strongly Agree. The scale does not contain any reverse-coded items. The ASWBS consists of four subdimensions: satisfaction with family relationships (4 items), satisfaction with one’s relationship with a significant other (4 items), life satisfaction (3 items), and positive feelings (4 items). In addition to subscale scores, a total subjective well-being score can be computed. The minimum and maximum possible scores are 15 and 60, respectively, with higher scores indicating greater levels of subjective well-being among adolescents. The scale accounts for 61.64% of the total variance. The overall internal consistency of the ASWBS is high, with a Cronbach’s alpha coefficient of 0.86 [68]. In the current study, the internal consistency of the ASWBS was calculated as 0.91, reflecting excellent reliability. Example items include the following: Item 7: “I am generally cheerful.”; Item 13: “I am close to the people I love.”

### 2.4. Procedure and Intervention

This study employed an experimental design involving pre-test, post-test, and follow-up assessments with experimental and control groups to evaluate the effectiveness of a mindfulness-based intervention program developed to reduce social media burnout and enhance subjective well-being among adolescents. The six-week duration was selected based on prior school-based intervention research, which emphasizes the feasibility and effectiveness of short-term, structured programs for adolescent populations [48,49,50]. A six-session format was deemed sufficient to introduce and consolidate key psychological competencies—such as emotion regulation, present-moment awareness, and self-compassion—without disrupting academic obligations.

**Figure 1:**
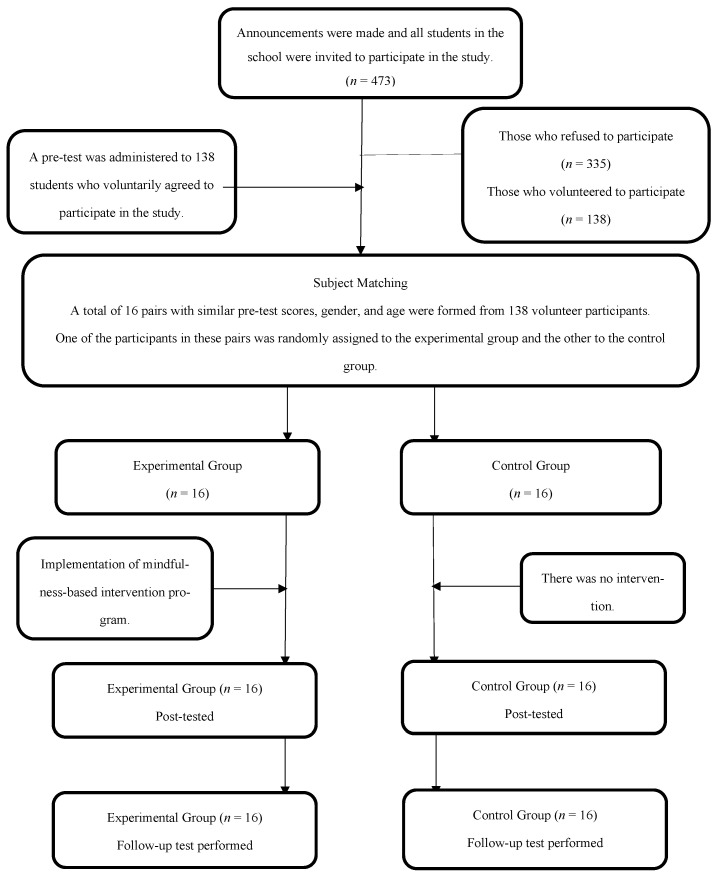
A flowchart of the process.

To ensure procedural rigor and transparency, the research process was conducted in seven clearly defined phases: (1) institutional approval, (2) participant recruitment, (3) group allocation, (4) baseline assessment, (5) intervention delivery, (6) post-intervention assessment, and (7) 30-day follow-up evaluation. This structured sequence allowed for the systematic evaluation of both short-term improvements and sustained effects—two key outcomes when assessing the impact of school-based psychological interventions targeting adolescents’ behavioral and emotional functioning. The overall research framework is depicted in Figure 1, and provided the foundation for methodological consistency across all stages of the study.

Initially, a public high school located in western Türkiye was contacted, and meetings were held with the school administration and the school counselor to explain the aim, scope, and procedures of the study in detail. Upon obtaining the necessary approval, information about the study was provided to 473 students enrolled at the school, and a total of 138 students expressed their voluntary willingness to participate by completing an online application form. The SMBS and the ASWBS were administered to these students to collect pre-test data. Based on their scores, as well as age and gender information, 16 matched pairs of participants with similar characteristics were created. Within each pair, one student was randomly assigned to the experimental group and the other to the control group using a lottery method. As a result, both groups consisted of 16 adolescents (9 females, 7 males), matched in terms of age and gender distribution. To determine whether the experimental and control groups were comparable at baseline, a Mann–Whitney U test was conducted. The results indicated no significant differences between the groups in either social media burnout (*p* = 0.83) or subjective well-being (*p* = 0.94) scores. This matching and allocation strategy was used to minimize group imbalance and increase internal validity within the constraints of a naturalistic school setting.

To ensure that the study was conducted in accordance with ethical principles, both the adolescents and their parents were fully informed about the purpose, the procedures, and their rights as participants. Written informed consent forms were obtained from all the participants and their legal guardians. It was explicitly stated that participants could withdraw from the study at any stage without any consequences. Prior to the commencement of data collection, ethical approval was obtained from the Ethics Committee of the university with which the researchers are affiliated. The study was conducted in compliance with the ethical standards outlined in the Declaration of Helsinki, emphasizing participant protection and data confidentiality.

Following this approval, the mindfulness-based intervention program was implemented with the experimental group over the course of six weeks, with one session conducted each week. Before initiating the intervention, individual meetings were held with each student in the experimental group to explain the session content and to collaboratively schedule the program in a way that minimized disruption to their academic activities. The sessions were strategically held during lunch breaks to address feasibility concerns within a school-based setting. This timing ensured accessibility and minimized conflicts with academic lessons, thereby increasing participation and program adherence.

The intervention program was conducted in weekly sessions lasting 40 to 50 min, following a structured format (see Table 1). Each session was delivered according to a consistent framework that included the following components: a review of the previous session (except for the first session), discussion of homework assignments (except for the first session), a warm-up activity, exploration of the weekly core theme, implementation of group activities, session summary, and assignment of homework for the following week (except for the final session). In the first session, participants engaged in introductory activities aimed at building rapport and raising initial awareness of their social media habits. The second session addressed the influence of social media interactions on thoughts, emotions, and behaviors. The third session focused on developing the ability to tolerate distressing emotions, while the fourth introduced practices to enhance present-moment awareness and attentional control. In the fifth session, participants created personalized digital detox plans, and the final session emphasized the development of self-compassion skills and included an overall evaluation of the intervention process. Each session integrated a multimodal approach, including psychoeducational instruction, mindfulness exercises, creative writing tasks, and group discussions. Although all components were present in every session, the psychoeducation and mindfulness practices typically occupied the largest portion of the session (approximately 60%), followed by reflective writing (20%) and group sharing (20%). The full intervention was facilitated by the same researcher to ensure consistency in group dynamics and program delivery.

All the adolescent participants in the experimental group attended at least five of the six sessions, and any missed sessions did not include the first or final sessions, which were considered critical to the structure of the program. No participants dropped out or requested to withdraw during the intervention. The control group did not receive any intervention throughout the study; data were collected only at the pre-test, post-test, and follow-up phases. To address potential placebo or Hawthorne effects, the control group was not given any alternative structured activity, but their engagement in the study was maintained through neutral interactions during each data collection phase. This approach limited the risk of expectation bias while preserving ecological validity.

To evaluate the effectiveness of the intervention program, both the experimental and control groups completed the SMBS and the ASWBS at three time points. The pre-test was administered to both groups prior to the commencement of the intervention to establish baseline levels. The post-test was conducted immediately after the six-week intervention to assess short-term changes in social media burnout and subjective well-being. A follow-up assessment was carried out 30 days after the completion of the program to evaluate its long-term effects. These three waves of assessment provided a comprehensive framework for examining both the immediate impact and the sustained outcomes of the mindfulness-based intervention. This longitudinal structure allowed for the assessment of treatment durability, which is often overlooked in brief school-based programs.

Given the relatively small sample size and the assumption that normal distribution cannot be reliably expected in small samples, non-parametric statistical methods were employed in the data analysis. The post-test scores of the experimental and control groups were compared using the Mann–Whitney U test. Within-group differences between pre-test and post-test scores were analyzed using the Wilcoxon Signed-Rank Test. The follow-up test results were also evaluated using the Wilcoxon Signed-Rank Test. The selection of these tests was guided by the methodological literature emphasizing their appropriateness for small-N designs and ordinal data structures. This structured analytic approach enabled a systematic, consistent, and reliable evaluation of both the short-term and long-term effects of the mindfulness-based intervention on adolescents’ social media burnout and subjective well-being.

### 2.5. Data Analysis

In this study, the pre-test, post-test, and follow-up scores for social media burnout and subjective well-being obtained from adolescents in the experimental (*n* = 16) and control (*n* = 16) groups were analyzed using SPSS 22.0 software. The literature emphasizes that in experimental studies—particularly those involving small sample sizes—the assumption of normality is often not realistically met, and in such cases, non-parametric statistical methods are recommended [69]. Accordingly, non-parametric analyses were employed for the evaluation of the research data. Although inferential analyses were based on non-parametric procedures due to the conservative assumption of non-normality, mean and standard deviation values were retained in the descriptive statistics for the purpose of sample characterization. This approach is commonly used in behavioral sciences to provide a familiar summary of group profiles, especially when reporting demographic and baseline characteristics.

To assess whether the experimental and control groups were statistically equivalent prior to the intervention, pre-test scores from the SMBS and the ASWBS were compared using the Mann–Whitney U test. The results indicated no significant differences between the groups at baseline (*p* > 0.05), confirming the initial comparability of the two groups. This finding supports the internal validity of the experimental design and suggests that the changes observed at the end of the intervention process can be attributed to the effects of the mindfulness-based intervention itself.

To evaluate the effectiveness of the intervention program, the post-test scores of the experimental and control groups were analyzed using the Mann–Whitney U test. In addition, within-group changes were assessed by comparing pre-test and post-test scores using the Wilcoxon Signed-Rank Test. To examine the sustainability of the intervention effects, the follow-up scores from the experimental group were also compared with their post-test scores using the same test.

To calculate effect sizes for non-parametric analyses, the formula proposed by Fritz et al. [70] was employed: *r* = *z*/√*N*. This approach is widely used in small-sample designs where tests such as the Mann–Whitney U test and Wilcoxon Signed-Rank test are appropriate. Cohen’s [71] criteria were used to interpret the magnitude of the effect sizes, with *r* values around 0.10 considered small, those around 0.30 considered moderate, and those around 0.50 considered large.

All statistical analyses were conducted using SPSS version 22.0, with a significance level set at 0.05. This systematic and rigorous analytical process enabled careful evaluation of the effects of the mindfulness-based intervention program on social media burnout and subjective well-being, thereby enhancing the reliability and validity of the findings.

## 3. Results

The findings regarding the effects of the mindfulness-based intervention program on adolescents’ social media burnout and subjective well-being levels are presented in Table 2. As shown in Table 2, a comparison of pre-test and post-test scores within the experimental group revealed a statistically significant decrease in social media burnout in favor of the post-test (*z* = −3.528, *p* < 0.05, *r* = 0.882), as well as a significant increase in subjective well-being (*z* = −3.526, *p* < 0.05, *r* = 0.881). These results indicate that the intervention program was highly effective in reducing social media burnout and enhancing subjective well-being among the adolescents in the experimental group. Accordingly, hypotheses H_1_ and H_2_ are supported.

**Figure 2:**
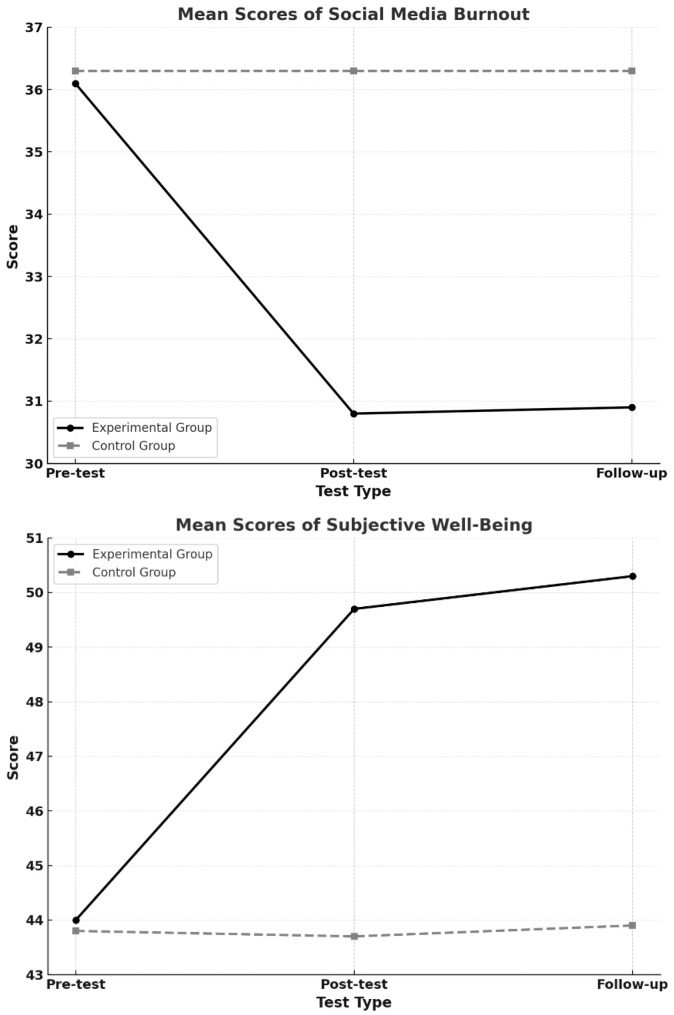
Score changes in groups according to intervention process.

In contrast, the comparison of the pre-test and post-test scores within the control group—who did not receive any intervention—showed no statistically significant changes in either social media burnout (*z* = −0.289, *p* = 0.773, *r* = 0.072) or subjective well-being (*z* = −0.322, *p* = 0.748, *r* = 0.080). These findings suggest that the control-group participants did not experience any notable change in these variables over time that could be attributed to natural developmental processes or external influences (see Figure 2).

When the post-test scores of the experimental and control groups were compared (see Table 2), the results indicated that the experimental group had significantly lower levels of social media burnout than the control group (*U* = 26.500, *p* < 0.05, *r* = 0.683). This finding supports a large effect of the intervention program in reducing social media burnout, thus confirming hypothesis H_3_. Similarly, the experimental group’s post-test scores for subjective well-being were significantly higher than those of the control group (*U* = 59.000, *p* < 0.05, *r* = 0.461), providing evidence for the substantial effectiveness of the mindfulness-based intervention in enhancing subjective well-being. Accordingly, hypothesis H_4_ is supported.

To assess the long-term effects of the intervention, a comparison of the experimental group’s post-test and follow-up scores revealed no statistically significant differences in social media burnout (*z* = −0.431, *p* = 0.666, *r* = 0.166) or subjective well-being (*z* = −1.581, *p* = 0.114, *r* = 0.395), as shown in Table 2. These results suggest that the positive outcomes achieved through the intervention were maintained after 30 days, indicating sustained benefits and lasting effects (see Figure 2). Therefore, hypothesis H_5_ is supported.

In contrast, comparisons between the post-test and follow-up scores of the control group—who received no intervention—showed no significant changes in either social media burnout (*z* = −0.122, *p* = 0.903, *r* = 0.030) or subjective well-being (*z* = −0.552, *p* = 0.581, *r* = 0.138). These findings indicate that the control-group participants did not experience any notable changes in these variables over time, reinforcing the conclusion that the observed effects in the experimental group were attributable to the intervention itself (see Table 2 and Figure 2).

## 4. Discussion

This study found that a mindfulness-based psychoeducational intervention program was effective in reducing social media burnout and enhancing subjective well-being among adolescents. According to the results, participants in the experimental group exhibited a significant decrease in social media burnout scores and a significant increase in subjective well-being scores following the intervention. Moreover, these positive outcomes were sustained in the follow-up assessment conducted 30 days after the completion of the intervention. The absence of similar changes in the control group further supports the conclusion that the observed improvements were attributable to the intervention itself. These findings reinforce the core hypothesis of the present study, which was grounded in a multi-theoretical framework integrating mindfulness, the ACT, and positive psychology perspectives.

These findings are largely consistent with prior research indicating that mindfulness-based interventions can reduce stress [72,73] and burnout [74,75], enhance emotion regulation [48,76], and improve subjective well-being [77,78]. However, the present study extends these findings by demonstrating that mindfulness practices can also alleviate a specific form of digital-age distress—social media burnout—which remains understudied in the intervention literature. In this respect, the study advances the existing literature, which has predominantly focused on general stress reduction [79,80], by showing that such interventions can also address specific forms of digital exhaustion. This contribution highlights the potential of mindfulness practices not only in managing traditional psychological stress, but also in coping with the distinctive psychological pressures of digital environments.

Furthermore, this study demonstrates that social media burnout can be effectively managed not only by reducing screen time or taking temporary breaks from social media [81,82], but also through practices that enhance mental and emotional awareness regarding the qualitative nature of social media engagement. Moving beyond the dominant intervention strategies in the literature that focus on “time restriction” or “social media abstinence” [83,84], the present findings highlight the effectiveness of a psychological model that targets individuals’ relational and cognitive patterns toward digital platforms. This perspective aligns with contemporary scholarship suggesting that sustainable behavioral change in the digital age should be grounded in internal awareness rather than imposed external restrictions [85,86,87]. In this context, the current intervention is theoretically rooted in ACT’s emphasis on value-based behavioral flexibility and in positive psychology’s conceptualization of well-being as a skillset that can be intentionally cultivated through reflective, strengths-based practices [25,57].

The observed improvements in subjective well-being further indicate that mindfulness-based interventions contribute not only to symptom reduction, but also to the enhancement of life satisfaction and positive affect, in alignment with previous models emphasizing the role of internal agency, self-determination, and meaning-making in well-being [88]. This outcome is consistent with the positive psychology framework proposed by Seligman and Csikszentmihalyi [57], which emphasizes the cultivation of meaning, engagement, and positive emotions as sustainable pathways to greater well-being. Moreover, the integration of present-moment awareness, self-compassion, and distress tolerance strategies into the intervention structure likely fostered deeper engagement with internal experiences, which, in turn, contributed to sustained gains in well-being beyond the intervention period.

One of the original contributions of this study to the literature lies in its systematic examination of the relationship between social media burnout and subjective well-being within the framework of an experimental intervention. While most prior studies have explored this relationship at a correlational level [89,90,91], the current research provides preliminary evidence suggesting a potential causal relationship between reduced burnout and enhanced well-being, as observed through an empirically tested mindfulness-based intervention. Although the quasi-experimental design does not allow for definitive causal inference, as is possible in randomized controlled trials, the matched-pairs structure and the use of pre-, post-, and follow-up assessments strengthen the internal validity of the findings. Furthermore, the inclusion of a follow-up assessment distinguishes this study from many existing short-term interventions by offering additional insight into the sustainability of the program’s effects over time [52,61]. Accordingly, the study offers valuable insights into how targeted psychological interventions may contribute to improvements in both burnout and well-being outcomes over time.

The findings also offer important insights from the perspective of the Turkish cultural context. In collectivist societies like Türkiye, individuals often show heightened sensitivity to social expectations, familial approval, and social comparison [40]. In such cultural environments, external validation mechanisms embedded in social media platforms may exert stronger pressures on adolescents’ self-worth perceptions [1,92], thereby intensifying the psychological consequences of social media burnout [67,93]. In response to these cultural dynamics, the intervention was intentionally designed to incorporate culturally salient values and concerns specific to Turkish adolescents. These adaptations included the integration of themes such as familial expectations, collectivist identity orientations, and academic achievement pressure into session discussions, activities, and reflective exercises. For instance, in Session 2 (on digital self-perception), group discussions were facilitated around how likes and comments on social media may reflect or conflict with adolescents’ perceived responsibilities toward their families. In Session 5, during the construction of digital detox plans, participants were encouraged to reflect on how family interactions could serve as supportive alternatives to passive screen engagement. In addition, culturally familiar metaphors (e.g., the culturally resonant concepts of “not bringing shame upon the family” or “not disappointing one’s family”) were used to help students connect mindfulness practices with culturally meaningful values. Session prompts and examples were also linguistically and thematically adapted to reflect the relational norms and collective priorities typical in Turkish youth culture. Against this backdrop, the study’s demonstration that social media burnout can be reduced and subjective well-being enhanced among Turkish adolescents underscores the importance of culturally sensitive, school-based interventions [94]. Particularly in cultural settings where academic achievement and social prestige are highly emphasized [40,95], the development of mindfulness-based skills may serve as a critical strategy to help individuals cope more effectively with the psychological pressures of digital environments.

In conclusion, this study makes a significant and multifaceted contribution to the literature by presenting an empirically supported, theoretically grounded, and culturally responsive intervention model that effectively mitigates social media burnout and enhances subjective well-being in adolescents. Beyond the positive outcomes observed, the study advances the field in several important ways. The intervention is built upon an integrative theoretical framework that synthesizes mindfulness, the ACT, and positive psychology, thus providing a comprehensive response to the unique psychological challenges of the digital age. Methodologically, the study addresses the need for ecologically valid school-based interventions by employing a quasi-experimental matched-pairs design with procedural fidelity. Conceptually, it redefines the scope of digital well-being interventions by moving beyond time-restriction strategies and instead emphasizing internal awareness, emotional flexibility, and value-based action. Collectively, these contributions deepen our understanding of how structured psychological interventions can sustainably enhance emotional resilience and promote adaptive engagement with digital technologies among adolescents.

## 5. Limitations and Implications

This study provides preliminary yet compelling evidence that a structured mindfulness-based intervention can effectively reduce social media burnout and enhance subjective well-being among adolescents. The observed improvements in the experimental group, which were maintained at the 30-day follow-up, suggest that mindfulness practices can serve as developmentally appropriate and contextually adaptable tools to mitigate the psychological toll of digital engagement among youth.

Nevertheless, several limitations must be acknowledged. First, the relatively small and demographically homogeneous sample—consisting of 32 students from a single public high school in Türkiye—limits the generalizability of the findings to more diverse populations. In particular, the urban location of the school may have introduced contextual biases, as adolescents in urban settings often differ from their rural counterparts in terms of digital access, social media usage patterns, and exposure to academic and familial pressures. These environmental factors may have shaped both the manifestation of social media burnout and the responsiveness to mindfulness-based strategies. Second, the study relied exclusively on self-report instruments, which may be susceptible to social desirability or recall bias, and did not include multi-informant assessments such as teacher ratings or behavioral observations. Future studies should consider incorporating objective behavioral metrics, such as digital activity logs or screen-time tracking applications, to complement self-report data and more accurately capture participants’ patterns of social media use and engagement. Third, the follow-up period was restricted to 30 days, offering only a limited view of the long-term sustainability of the intervention effects. Finally, the quasi-experimental design, although strengthened through matched-pairs randomization, does not offer the same level of causal inference as a fully randomized controlled trial.

To address these constraints, future research should aim to replicate this intervention model across culturally diverse and demographically varied adolescent populations. Expanding sample sizes and implementing multi-site studies will enhance external validity. Moreover, future studies should consider including qualitative feedback from participants to better capture the mechanisms of change and the perceived relevance of intervention content. Longitudinal follow-up assessments (e.g., 3 or 6 months post-intervention), along with digital behavior markers or passive sensing technologies (e.g., social media usage metadata or app interaction patterns), would also strengthen the evidence base for sustained impact. Importantly, future models should explore systemic adaptations that integrate the perspectives of parents, teachers, and peers to maximize scalability and ecological validity.

The theoretical implications of this research are substantial. By integrating mindfulness, the ACT, and positive psychology frameworks [96], the study advances a multidimensional model of psychological well-being in the digital age. Rather than promoting behavioral avoidance (e.g., screen time restriction), the intervention focuses on strengthening internal capacities such as psychological flexibility, emotional regulation, and values-based action—constructs known to predict long-term resilience and flourishing [97]. The demonstrated causal pathway from reduced social media burnout to improved well-being offers novel insights into the developmental processes through which adolescents can navigate digital stressors more adaptively.

From a practical perspective, the program’s structured, brief, and scalable format makes it well-suited for implementation in school-based mental health services. School counselors can deliver the intervention in small-group formats with minimal disruption to academic schedules. However, the feasibility of training school counselors in mindfulness-based approaches should also be acknowledged as a potential implementation barrier. While the intervention content is standardized and protocol-driven, effective delivery may require counselors to develop a foundational level of mindfulness practice and facilitation skills. Future dissemination efforts should therefore include accessible training modules, supervision opportunities, and supportive materials to build counselor competence and confidence. The content also aligns with preventive mental health priorities, offering a proactive strategy to address digital-age challenges before they escalate into clinical concerns. For educational policymakers, the findings highlight the importance of incorporating digital resilience, mindfulness, and emotional awareness into high school guidance curricula. Institutional investment in counselor training, time allocation, and resource support is crucial to ensure program fidelity and sustainability. In addition, future program adaptations should explore concrete strategies for incorporating teachers and caregivers into the intervention framework. For example, brief psychoeducational sessions can be developed for parents to help them support adolescents’ mindful technology use at home, while school staff can be trained to reinforce core concepts (e.g., emotion regulation, self-compassion) in classroom and extracurricular settings. Such multi-component models may enhance program impact through consistent reinforcement across school and family environments. Moreover, adapting key practices for use with teachers and caregivers may amplify impact through multi-level support systems.

In conclusion, this study introduces a theoretically informed and empirically supported mindfulness-based intervention that addresses a critical and understudied issue in adolescent mental health: the psychological consequences of social media burnout. By bridging theory and practice in a culturally sensitive, developmentally grounded manner, the study contributes to the growing body of research aimed at safeguarding adolescent well-being in an increasingly digitalized world.

## Figures and Tables

**Table 1:** Mindfulness-based intervention program for adolescents.

Topic	Description
Meet + Social Media Awareness	In the first session, participants engaged in introductory activities, during which group rules were collaboratively established and the overall structure of the program was explained. The primary aim of this session was to foster initial awareness of adolescents’ social media use habits. Each participant was provided with a “social media journal” and asked to monitor both the duration of their use and the emotional responses they experienced throughout the following week.
Thought–Emotion–Behavior Relationship	The second session focused on the emotional and cognitive impacts of social media interactions. Participants reflected on specific emotional reactions following particular posts or interactions and identified the accompanying cognitive responses. Through guided group discussions, the concepts of “authentic self” and “digital self” were explored and contrasted.
Staying with Emotions	This session aimed to strengthen participants’ capacity to cope with distressing emotions. Using mindfulness-based breathing exercises and an activity titled “a letter to a difficult emotion,” participants practiced staying present with challenging feelings without avoidance or suppression. Additionally, a brief mindful observation walk was conducted to support nonjudgmental awareness of emotions and thoughts.
Focus on the Present Moment	The fourth session focused on enhancing attention and present-moment awareness skills. Activities included a five-senses mindfulness exercise and mindful eating practice, both designed to ground attention in the here and now. The session concluded with a guided “mental journey” meditation, encouraging participants to observe their thoughts as transient mental events.
Digital Detox Plan	In this session, participants were guided to critically reflect on their social media habits and to develop personalized digital detox plans. Each adolescent set a goal to reduce their screen time over the following week and identified meaningful alternative offline activities. A group “digital detox contract” was created to promote accountability and collective commitment to the process.
Self-Compassion + Evaluation of the Process and Farewell	The final session was devoted to program evaluation and the reinforcement of self-compassion skills. Through the “letter of kindness to myself” activity, participants reflected on their personal growth and internalized the key practices from the program. Post-test assessments were conducted, and the group process was brought to a close with a meaningful shared reflection and farewell.

**Table 2:** Comparison of group means for pre-test, post-test, and follow-up test scores.

Variables	Experimental Group (*n* = 16)	Control Group (*n*= 16)	*U*	*p*	*r*
	*M* ± *SE*	*M* ± *SE*			
Social Media Burnout					
Pre-test	36.125 ± 2.705	36.312 ± 2.915	122.500	0.833	0.037
Post-test	30.812 ± 3.351	36.375 ± 3.384	26.500	0.000	0.683
Follow-up test	30.875 ± 2.849	36.375 ± 3.403	22.500	0.000	0.705
*z* (pre- × post-test)	−3.528	−0.289			
*p*	0.000	0.773			
*r*	0.882	0.072			
*z* (post- × follow-up test)	−0.431	−0.122			
*p*	0.666	0.903			
*r*	0.166	0.030			
Subjective Well-Being					
Pre-test	43.875 ± 7.805	43.750 ± 7.785	126.000	0.940	0.013
Post-test	49.750 ± 4.420	43.687 ± 7.409	59.000	0.008	0.461
Follow-up test	50.375 ± 4.048	43.812 ± 7.350	53.500	0.005	0.498
*z* (pre- × post-test)	−3.526	−0.322			
*p*	0.000	0.748			
*r*	0.881	0.080			
*z* (post- × follow-up test)	−1.581	−0.552			
*p*	0.114	0.581			
*r*	0.395	0.138			

Note: *U*, Mann–Whitney U test; *z*, Wilcoxon signed-rank test; *r*, effect size.

## Data Availability

Data will be provided upon request. The data are not publicly available due to potential to compromise the privacy of the participants.
